# Genetic dissection of the neuro-glio-vascular machinery in the adult brain

**DOI:** 10.1186/s13041-017-0345-4

**Published:** 2018-01-15

**Authors:** Gregory W. Kirschen, Rachel Kéry, Hanxiao Liu, Afrinash Ahamad, Liang Chen, Wendy Akmentin, Ramya Kumar, Joel Levine, Qiaojie Xiong, Shaoyu Ge

**Affiliations:** 1 Medical Scientist Training Program, Stony Brook, New York USA; 2 Department of Neurobiology & Behavior, SUNY Stony Brook, Stony Brook, NY 11794 USA; 3 School of Health Technology & Management, Stony Brook University, Stony Brook, NY 11794 USA

**Keywords:** Neurovascular coupling, Astrocyte, Canine adenovirus, Adeno associated virus, Adult hippocampal neurogenesis, Dentate granule cell

## Abstract

The adult brain actively controls its metabolic homeostasis via the circulatory system at the blood brain barrier interface. The mechanisms underlying the functional coupling from neuron to vessel remain poorly understood. Here, we established a novel method to genetically isolate the individual components of this coupling machinery using a combination of viral vectors. We first discovered a surprising non-uniformity of the glio-vascular structure in different brain regions. We carried out a viral injection screen and found that intravenous Canine Adenovirus 2 (CAV2) preferentially targeted perivascular astrocytes throughout the adult brain, with sparing of the hippocampal hilus from infection. Using this new intravenous method to target astrocytes, we selectively ablated these cells and observed severe defects in hippocampus-dependent contextual memory and the metabolically regulated process of hippocampal neurogenesis. Combined with AAV9 targeting of neurons and endothelial cells, all components of the neuro-glio-vascular machinery can be simultaneously labeled for genetic manipulation. Together, we demonstrate a novel method, which we term CATNAP (CAV/AAV Targeting of Neurons and Astrocytes Perivascularly), to target and manipulate the neuro-glio-vascular machinery in the adult brain.

**Electronic supplementary material:**

The online version of this article (10.1186/s13041-017-0345-4) contains supplementary material, which is available to authorized users.

## Introduction

The adult human brain comprises only about 2 % of the body’s weight, yet, it consumes nearly 20 % of its resting metabolic resources [14]. Studies employing in vivo functional neuroimaging have consistently demonstrated that the central nervous system exhibits a high metabolic demand [15, 54]. There is a complex interplay between neurons and the vasculature in the behaving animal [38, 64]. Yet, the mechanisms underlying this functional coupling remain poorly understood. This is due in part to a shortage of effective methods to specifically target the neurovascular coupling machinery.

The blood brain barrier, the interface between neurons and vessels, is formed by tight junctions of brain endothelial cells [22]. During development, the barrier is further wrapped by pericytes and astrocytes, which work together throughout life to ensure structural stability and functional vascular dynamics [13, 16, 55, 63]. Live imaging, ultrastructural and in vitro models show an intimate relationship between endothelial cells and mural cells at the barrier interface [7, 47, 56]. These cells control vital functions such as barrier permeability and nutrient/waste exchange [1, 59]. Astrocytes are key components of this multi-cellular complex, and neuronal stimulation can presumably activate these cells, leading to changes in the rate of blood flow [41]. Similarly, pharmacological activation or inhibition of astrocytes alters blood flow regulation, which in turn regulates nutrient and metabolic waste exchange in the brain [34, 41]. Astrocytes also orchestrate a major component of the brain’s adaptive response to vascular injury or ischemia, forming a protective glial scar and aiding in post-insult regeneration and repair [6, 35]. In turn, insults that disrupt oxygen or nutrient supply to the brain can often alter the physiology of astrocytes [23, 24, 27]. Despite this progress in understanding the structure and potential function of the neurovascular coupling unit, our knowledge of how exactly the individual components of the coupling machinery work in concert remains rudimentary. To advance our understanding and expand the translational potential of blood brain barrier physiology, it is necessary to systematically dissect the various parts and determine how they interact.

In this study, we established a novel method to specifically target and manipulate the neuro-glio-vascular machinery of the adult brain. We carried out a viral labeling screen in adult animals, and identified Canine Adenovirus 2 (CAV2) as a tool to non-invasively label perivascular astrocytes preferentially. Together with intravenous adeno associated virus serotype 9 (AAV9) injection, which, when delivered intravenously, can specifically label endothelial cells [62], the components of the neuro-glio-vascular machinery can all be readily targeted for simultaneous genetic manipulation. Using this approach, we uncovered several novel heterogeneous features of perivascular astrocytes as well as a crucial role in the regulation of adult hippocampal neurogenesis, an activity-dependent metabolic brain process [5]. Together, our study reports a three-pronged viral method capable of genetically targeting the neuro-glio-vascular machinery in the adult brain.

## Methods

### Surgeries and procedures

All surgeries and experimental procedures were approved by the Stony Brook University Animal Use Committee and followed the guidelines of the National Institutes of Health. Prior to all surgeries and intravenous injections, mice were anesthetized with ketamine/xylazine cocktail (200 mg/kg, i.p.), and placed on a 38 degree C heating pad to recover. Following stereotaxic surgeries, mice were administered buprenorphine HCl (0.05 mg/kg i.p.) for immediate post-surgery analgesia.

### Mice

Experiments were conducted using 6- to 8-week-old male and female wildtype C57BL/6 mice (Charles River Laboratories), Ai14 (Gt(ROSA)26Sortm14(CAG-tdTomato)Hze) mice (The Jackson Laboratory), and inducible diphtheria toxin receptor (iDTR) (C57BL/6-Gt(ROSA)26Sortm1(HBEGF)Awai/J) mice (The Jackson Laboratory). All mice were housed in pairs and maintained on a 12 h light/dark cycle. All behavioral experiments were performed during the light cycle. Mice were provided ad libitum access to food and water.

### Viruses

Retrovirus and lentivirus production was performed as we described previously [21]. Adeno-associated virus 9 (AAV9)-*CAG-GFP* and AAV9-*CAG-Cre* were purchased from the University of North Carolina Vector Core. CAV2-Cre was purchased from Institut de Génétique Moléculaire de Montpellier (IGMM). Animals were sacrificed three-weeks post-injection. Tail vein injections were performed as previously described [18]. Intra-hippocampal viral injections (0.5 μl/injection site) were performed using stereotactic coordinates: 2.0 mm posterior to bregma, 1.6 mm lateral, 2.5 mm ventral, and 3.0 mm from bregma, 2.6 mm lateral, 3.2 mm ventral. Intra-thalamic injections (0.5 μl/injection site) were performed using stereotactic coordinates: 3.2 mm posterior to bregma, 2.0 mm lateral, 2.7–3.1 mm ventral. Intra-striatal injections (0.5 μl/injection site) were performed using stereotactic coordinates: 1.7 mm posterior to bregma, 3.15 mm lateral, and 2.1–2.5 mm ventral.

### Inducible diphtheria toxin receptor-mediated cell ablation

iDTR and WT mice were administered DT (0.125 μg/g dissolved in sterile saline, i.p., q.d.) or an equal volume of sterile saline i.p., q.d. in the morning for 3 consecutive days, similar to what has been previously reported [11].

### Arterial labeling with Alexa Fluor 633 Hydrazide

We conducted arterial labeling with intravenously-delivered Alexa Fluor 633 Hydrazide as previously described [52]. Briefly, mice were anesthetized with ketamine/xylazine cocktail (200 mg/kg, i.p.) and injected with Alexa fluor 633 hydrazide (1 mg/kg). After 4–5 h, the mice were then deeply anesthetized with urethane (200 μg/g) and perfused transcardially with PBS and then 2% PFA. Brains were sectioned on a vibratome into 60 μm-thick coronal sections and immediately imaged on an Olympus FLV1000 confocal microscope.

### Tissue processing, imaging, and quantification

Mice were deeply anesthetized with urethane (200 μg/g) and perfused transcardially with PBS and then 4% PFA. Brains were removed, fixed overnight in 4% PFA, transferred to a 30% (*w*/*v*) sucrose solution, and stored at 4 °C until sectioning. Brains were sectioned into 60 μm-thick coronal sections covering the entire anterior–posterior axis of the DG. Immunohistochemistry was performed by blocking sections in 1% donkey serum in PBS 0.025% Triton for 1 h at room temperature (after incubation in 2 N HCl for 25 min at 37 °C for BrdU only) and then switched to incubation in primary antibody, BrdU (rat polyclonal antibody, 1:1000; Abcam), GFP (rabbit polyclonal antibody, 1:1000; Sigma-Aldrich), DCX (goat polyclonal, 1:1000; Santa Cruz), GFAP (rabbit polyclonal, 1:500; Dako), RFP (rabbit poyclonal, 1:500, Rockland), Nestin (chicken polyclonal, 1:250; Novus), CC1 (mouse monoclonal, 1:500; Millipore), Iba1 (rabbit polyclonal, 1:500; Wako), PDGFRα (mouse monoclonal, 1:500; Santa Cruz), NeuN (mouse monoclonal, 1:500; Millipore), CAR (mouse monoclonal, 1:250; Santa Cruz), CD31 (rat polyclonal, 1:500; BD Pharmingen) with overnight shaking at 4 °C.

Sections were then switched to the appropriate secondary antibody for 3 h with shaking at room temperature. The following antibodies were used: Alexa Fluor 488-conjugated donkey anti-rat antibody (1:1000; Abcam), Alexa Fluor 594-conjugated donkey anti-rat antibody (1:1000; Abcam), Alexa Fluor 488-conjugated donkey anti-rabbit antibody (1:1000; Jackson Laboratories), and Alexa Fluor 488-conjugated donkey anti-goat (1:1000, Abcam) for 3 h shaking at room temperature.

Confocal images were obtained on an Olympus FLV1000 confocal microscope. DCX+, Ki67+, Nestin + and GFAP+ cells were counted using a stereological unbiased systematic sampling approach of 60 μm *Z*-stacked images (175 μm 175 μm in *x* and *y* planes) with 2 μm guard zones, as we previously described [31]. Images were 3-D reconstructed in Imaris Scientific 3D/4D Processing & Analysis Software (Bitplane). Branch analysis was conducted in ImageJ using the NeuronJ plugin. Distances between GFAP+ cells and endfoot contacted CD31+ cells was conducted by drawing a straight line from the center of the GFAP+ cell nucleus to the point of contact for each process.

For cortical astrocyte density measurements, cells in the molecular layer (Layer I) of cerebral cortex were analyzed, as these astrocytes exhibit many filamentous GFAP+ cells under normal conditions [4, 42].

For the c-Fos experiment, mice were left undisturbed in their home cages for 4 h prior to transcardiac perfusion to allow baseline c-Fos in the dentate gyrus to be measured.

CAR fluorescence intensity profiles were analyzed in ImageJ using gray levels along astrocyte somata and processes as previously described [50]. Heat maps of relative fluorescence intensity along astrocytic somata and processes were generated in Matlab using a 1-D data interpolation function. Blood vessel density was quantified in ImageJ using the area selection function.

### Electron microscopy tissue preparation

Animals were perfusion fixed with a mixture of cold 2% paraformaldehyde +0.1% glutaraldehyde in phosphate buffer (PB), pH 7.4. After perfusion the brains were removed and post fixed for several hours. Brains were then sectioned on a Vibratome at 50–60 μm in cold PB and stored at 4^o^ C overnight. The next day, the sections were post fixed for 1 h with osmium tetroxide (1%, 0.1 M PB), rinsed, en bloc stained with 1% uranyl acetate, rinsed, dehydrated through an ascending series of ethanols and embedded in Durcupan epoxy resin. Sections were sandwiched between sheets of ACLAR and cured at 60 °C for 48 h.

Blocks of tissue containing hippocampus were sectioned at 60–70 nm with a Reichert-Jung Ultracut E ultramicrotome. Ultrathin sections were mounted on Formvar-coated, nickel-slot grids.

Sections were postembedding immunogold labeled within 24 h of sectioning by using a modification of the protocol of Phend et al. [49]. Grids were then air dried, stained with uranyl acetate and lead citrate, and examined at 80 kV with a JEOL 1200 EX electron microscope (JEOL, Peabody, MA).

### Behavioral apparatuses and procedures

The open field test was conducted as follows. Briefly, mice were placed in an open arena (50 cm^2^ opaque box) lacking objects for 5 min. Animal paths were recorded using EthoVision XT video tracking software. Contextual fear conditioning and discrimination were performed as we previously described [21]. Mice were placed in a fear-conditioning chamber consisting of transparent front and back walls, stainless steel sidewalls and a stainless steel shock grid floor (18 × 18 × 30 cm, Coulbourn). In fear context A, 70% ethanol was used to remove any odor for the context before each experiment. During training in context A, mice were allowed to explore the context for 150 s before receiving a 3-s foot shock (0.5 mA). Mice were removed from the conditioning chamber 30 s after the foot shock and transferred back to their home cages. A probe test for contextual fear memory was conducted 24 h after training. Mice were re-exposed to context A without tone or foot shock, and freezing levels during a 5-min period were measured. A probe test for contextual discrimination was conducted in the afternoon of the same day by placing mice into a novel context B (16 cm diameter cylindrical environment, cross-hatched stainless steel floor grid) and freezing levels were recorded during for a 5-min period. Mice movements were recorded using Freeze Frame software, and freezing levels were analyzed by Freeze View so ware with 1-s minimum bout duration.

### Quantification and statistical analysis

Data were analyzed with independent- and paired-samples *t* tests, two-way ANOVA, one-way ANOVA followed by post hoc the least significant difference (LSD) test, one-way ANOVA with repeated measures followed by post hoc paired *t* tests. Two-tailed α values of 0.05 were considered the cutoff for statistical significance. *n* represents the number of animals. All statistics can be found in the figure legends.

### Key resources

**Table:** 

REAGENT or RESOURCE	SOURCE	IDENTIFIER
Antibodies		
anti-Ki67 rabbit polyclonal	Novocastra	NCL-Ki67p
anti-GFP rabbit polyclonal	Sigma-Aldrich	G1544-100UG
anti-GFAP rabbit polyclonal	Dako	#Z0334
anti-DCX goat polyclonal	Santa Cruz	sc-8066
anti-RFP rabbit polyclonal	Rockland	600–401-379
anti-Nestin chicken polyclonal	Novus	NB100–1604
anti-CC1 mouse monoclonal	Millipore	OP80-100UG
anti-Iba1 rabbit polyclonal	Wako	019–19,741
anti-PDGFRα mouse monoclonal	Santa Cruz	sc-398,206
anti-NeuN mouse monoclonal	Millipore	MAB377
anti-CAR mouse monoclonal	Santa Cruz	sc-373,791
anti-CD31 rat polyclonal	BD Pharmingen	550,274
anti-Cre recombinase goat polyclonal	Abcam	Ab24607
Anti-c-Fos rabbit polyclonal	Santa Cruz	Sc-52
Virus Strains		
CAV2-*CMV-Cre*	Institut de Génétique Moléculaire de Montpellier (IGMM)	Contact: CAV.2@biocampus.cnrs.fr
AAV9-*CAR-GFP*	Customized from University of Pennsylvania Vector Core	Contact: vector@mail.med.upenn.edu
AAV9-*CAG-Cre-GFP*	University of Pennsylvania Vector Core (commercially available)	AV-9-ALL854
AAV9-*CAG-GFP*	University of Pennsylvania Vector Core (commercially available)	AV-9-ALL851
Retro-*Ubi-GFP*	Generated in-house	
Lenti-*Ubi-GFP*	Generated in-house	
Lenti-*GFAP-GFP*	Generated in-house	

## Results

### Heterogeneity and commonalities between perivascular astrocytes in the adult brain

**Fig. 1:**
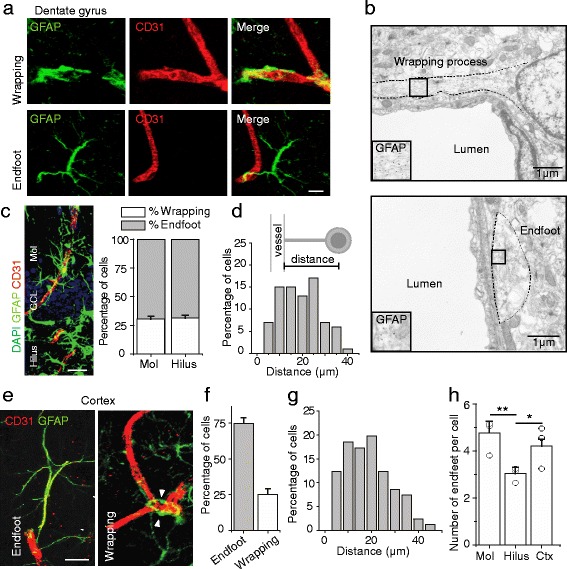
Two types of glio-vascular units in the adult brain. **a** Representative images of astrocytes (GFAP+ cells) exhibiting wrapping-type and endfoot-type morphologies with respect to neighboring blood vessels (CD31+ cells) in the Molecular layer (Mol) of the dentate gyrus. **b** Representative immunogold-stained electron micrographs of blood vessels contacted by a GFAP+ cell wrapping process (top) and a GFAP+ cell endfoot (bottom). Black dots show GFAP staining. Images are taken from the Mol of the dentate gyrus. **c** Cross section through the laminae of the dentate gyrus, showing astrocytes and associated blood vessels (Molecular layer, Mol; Granule Cell Layer, GCL; Hilus). On the right is a plot of the percentage of astrocytes exhibiting the wrapping type morphology in the Mol and Hilus. Two-tailed unpaired t-test, *P* = 0.966. **d** Histogram of distances between the center of the astrocytic nucleus and contacted endothelial cells. The inset depicts an astrocyte contacting a vessel via an endfoot and the calculated distance. **e** Representative images of wrapping-type and endfoot-type astrocytes in the auditory cortex. **f** Shown is a plot of the proportion of wrapping-type and endfoot-type astrocytes in primary auditory cortex. **g** Histogram of distances between the center of the astrocytic nucleus and contacted endothelial cells in primary auditory cortex. **h** Plot of the number of endfeet per astrocyte in different regions. One-way ANOVA *F*(3,93) = 5.587, *P* = 0.005. Post-hoc LSD tests, Mol vs Hilus, *P* = 0.001; Mol vs Ctx, *P* = 0.304; Hilus vs Ctx, *P* = 0.033. The scale bars are 10 μm. Data are plotted as mean +/− SEM. * indicates *P* < 0.05, ** indicates *P* < 0.01. *n* = 3–5 animals per experiment

Neuronal activation promotes local capillary blood flow [3], facilitating functional hyperemia to orchestrate circuit activity, information processing, and behavior [53, 61]. While the importance of neurovascular coupling is widely appreciated, the mechanisms underlying this interplay remain poorly understood. Brain astrocytes form endfeet that directly contact micro-vessels [1], and can surround neuronal synapses [17]. By forming a physical connection with both blood vessels and neurons, astrocytes may play an essential role in propagating neuronal activity to regulate vascular activity. We therefore first sought to better understand their anatomical features, focusing on their relationships to microvessels. We selected the hippocampal dentate gyrus, which contains a compact laminal structure of neuronal cell bodies, dendrites, and axons. We stained adult brain sections with an antibody directed against glial fibrillary acidic protein (GFAP*)* [8]. To mark vessels, we co-stained the sections with an antibody for CD31, an endothelial cell marker. Based on their association with the vessels, we classified the astrocytes into two types: 1) those that wrapped their somas and processes around vessels (defined as wrapping-type), and 2) those that contacted vessels via endfeet (defined as endfoot-type) (Fig. 1a). We confirmed the physical association between astrocytes and vessels using immuno-electron microscopy as shown in Fig. 1b. These two cell types together represented more than 90% of astrocytes identified by GFAP staining. The remaining minority of astrocytes not found to be contacting vessels may have resulted from disruptions in vessel contacts during tissue sectioning. We analyzed the distribution of wrapping- versus endfoot-type of astrocytes across the layers of the dentate gyrus. As shown in Fig. 1c, the ratio between these two types of cells was relatively uniform between the hilus and molecular layers (excluding the granule cell layer, which contains GFAP+ radial glia-like cells). We further analyzed the distances between vessels and the somata of astrocytes of the endfoot group, and found the median distance to be 22.8 μm, with no differences between layers (Fig. 1d).

Given the heterogeneity of hippocampal astrocyte-blood vessel contacts, we wanted to see whether these anatomical findings would generalize to other brain regions. Therefore, we analyzed quantitatively the two classes of astrocytes in the neocortex. Approximately three fourths of total astrocytes were the endfoot-type, with the remainder being wrapping-type, similar to the ratios observed in the dentate gyrus (Fig. 1e and f). The mean distance between endfeet and vessels was 20.7 μm (Fig. 1g), similar to that found in the dentate gyrus. We also analyzed the number of endfeet per astrocyte and found that while cortical astrocytes had a comparable number of endfeet per cell compared to the molecular layer of the DG, the hilus of the DG had significantly fewer (Fig. 1h). Astrocyte processes in the molecular layer were most highly ramified, while the diameter of endfeet across anatomical regions did not differ significantly (Additional file 1: Figure S1).

Taken together, our analyses showed that the cortex and hippocampus both exhibited two typical classes of astrocytes. Although there were many common features in the analyzed brain areas, astrocytes also showed some morphological differences. Whether this morphological heterogeneity translates to a functional difference, for instance whether hilar astrocytes represent a distinct subpopulation of astrocytes that regulate functional hyperemia differently, remains to be tested.

### Intravenous CAV2 preferentially labeled astrocytes in the adult brain

**Fig. 2:**
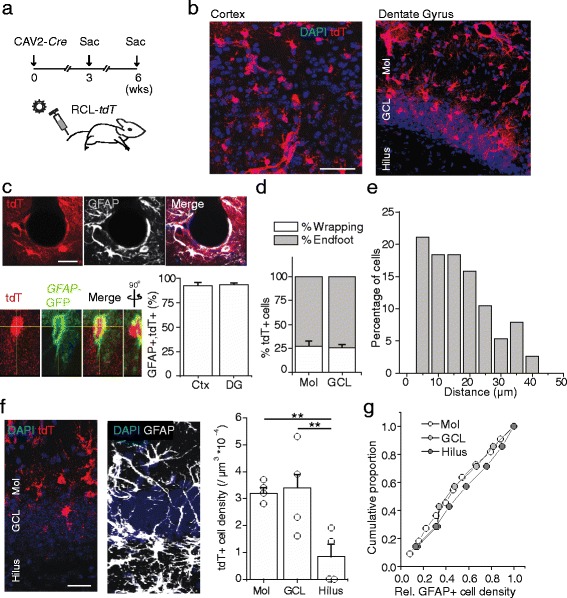
Preferential labeling of astrocytes via intravenous CAV2. **a** Experimental timeline of intravenous CAV2-*Cre* injection into RCL-*tdT* animals. **b** Coronal sections through cortex and dentate gyrus showing the distribution of tdT-expressing cells at 3 weeks after CAV2-*Cre* injection into RCL-*tdT* mice. The scale bar is 50 μm. **c** Co-localization between an anti-GFAP antibody and tdT three weeks after CAV2-*Cre* injection into RCL-*tdT* mice (top) The scale bar is 10 μm. Co-localization between tdT and a GFP reporter expressed by lentiviral-*GFAP-GFP*-infected cells (bottom left). Plot of the proportion of tdT cells that co-localized with GFAP in cortex and DG (bottom right). **d** Plot of the proportion of wrapping type tdT+ cells in the different laminae of the dentate gyrus. **e** Histogram of the distance distribution between tdT-expressing cells and contacted CD31+ cells in the dentate gyrus. **f** Representative images of the dentate gyrus displaying tdT and GFAP distribution in the different laminae (left). Plot of the density of tdT+ cells in the different layers of the dentate gyrus (right). One-way ANOVA *F*(2,39) = 11.57, *P* < 0.001. LSD test Mol vs GCL, *P* = 0.819; Mol vs Hilus, P < 0.001; GCL vs Hilus P < 0.001. **g** Plot of the relative distribution of astrocyte density in the three laminae of the dentate gyrus. One-way ANOVA *F*(2,18) = 2.71, *P* = 0.094. Data are plotted as mean +/− SEM. * indicates P < 0.05, ** indicates P < 0.01. Scale bars are 10 μm. *n* = 4 animals per experiment

The importance in neurovascular coupling and region-specific heterogeneity of astrocytes motivated us to search for a genetic tool to target this population of cells. We infused high titer lenti-, retro- and adeno-associated (AAV type 9) reporter viruses stereotaxically into the adult mouse brain. Because of the tight connection of astrocytes with vessels, we also performed tail vein injections. As shown in Additional file 1: Figure S2a and Additional file 1: Table S1, we observed little success in intravenous transgene delivery, although importantly, we were able to detect fluorescent signal in endothelial cells with AAV9 delivery, consistent with previous findings [51]. As expected, all viruses were able to target neurons when injected stereotaxically (Additional file 1: Figure S2a). Likewise, stereotaxic injection of CAV2 harboring the gene coding for *Cre recombinase* into the brain of adult *Cre* reporter mice whose cells flip in and express *tdTomato* (*tdT*) in the presence of Cre recombinase (RCL-*tdT*, [39]) robustly labeled neurons, consistent with previous findings [48] (Additional file 1: Figure S2b). Intriguingly, 3 weeks after intravenously delivering CAV2-*Cre*, we found labeled intraparenchymal cells that did not exhibit typical neuronal morphologies throughout the brain (Fig. 2a and b and Additional file 1: Figure S2a, c, d). To determine whether infectivity or expression of CAV2-delivered genes would expand over time, we analyzed brain sections 6 weeks post-injection (Fig. 2a). We noted no apparent differences in the pattern, density, or intensity of labeled cells at post injection of 6 weeks as compared to 3 weeks (Fig. 2b and Additional file 1: Figure S2c and d), suggesting the CAV2-mediated gene expression was relatively stable during this period.

To characterize the identity of these tdT+ cells, we immunostained brain sections for markers of the various cell types of the central nervous system. We found that in both the neocortex and hippocampus, almost all tdT+ cells were marked with GFAP, suggesting preferential labeling of astrocytes (Fig. 2c). By contrast, we observed no apparent co-localization with other glial markers including Nestin (predominantly staining radial glia-like cells), CC1 and PDGFRα (oligodendrocytes and oligodendrocyte precursors, respectively), peri-capillary NG2-positive cells (pericytes), or Iba1 (microglia) (Additional file 1: Figure S3a-e). Neither were these cells endothelial cells (Additional file 1: Figure S3f). We also did not observe co-localization with NeuN, a neuronal marker (Additional file 1: Figure S3g). To further confirm the GFAP expressing identity of marked tdT+ cells, we stereotaxically injected a lentivirus carrying GFP under the *GFAP* promoter. As expected, nearly all *GFAP*-driven GFP-expressing cells co-localized with intravenous CAV2-*Cre*-driven tdT (Fig. 2c).

We next analyzed the tdT+ cells as outlined in Fig. 1c and d. Approximately one fourth of labeled cells had a wrapping morphology and three fourths an endfoot morphology (Fig. 2d and Additional file 1: Figure S4a, b). Likewise, the distribution of distances between tdT+ cell bodies and CD31+ endothelial cells closely paralleled our findings from GFAP-expressing cells in Fig. 1a (Fig. 2e and Additional file 1: Figure S4c). These findings further confirmed the identity of tdT+ cells as astrocytes.

During the analysis, we noted in the analyzed brain regions that most astrocytes were tdT+, suggesting the broad labeling of this cell type by intravenous CAV2. However, in the hilus, we observed much fewer tdT+ cells (Fig. 2b and f). The lack of hilar labeling with CAV2 could not be explained by a lower density of astrocytes in this region, as indeed, the hilus contained an equal to slightly greater density of astrocytes as compared to the Mol and GCL at both 3 and 6 weeks post-injection (Fig. 2f and g and Additional file 1: Figure S2d, respectively). We did not see differences in the density of blood vessels across the three layers (Additional file 1: Figure S5), suggesting the difference in astrocyte labeling was not due to differences in vessel density. To verify that Cre recombinase was indeed present in infected tdT+ cells but not in tdT- hilar cells, we stained for Cre recombinase, and noted a pattern that closely resembled the tdT staining (Additional file 1: Figure S2e), indicating that hilar astrocytes lacked Cre recombinase.

The biased labeling in the dentate gyrus and broad labeling in other brain areas suggest a strategy to examine the function and heterogeneity of astrocytes. Together, the method we presented here, when combined with accessible chemo- and opto-genetic or traditional genetic approaches, will allow us to manipulate this population of cells to study the underlying mechanisms of astrocytic and vascular coupling. The astrocytic labeling presented in Fig. 2 can also be combined with other transgene delivery methods. For example, in Additional file 1: Figure S6, we demonstrated how intravenously-delivered CAV2 could be combined with stereotaxically- and intravenously-delivered AAV9 to simultaneously genetically target three components of the neuro-glio-vascular unit in vivo.

### Astrocytes express the Coxsackie adenovirus receptor

**Fig. 3:**
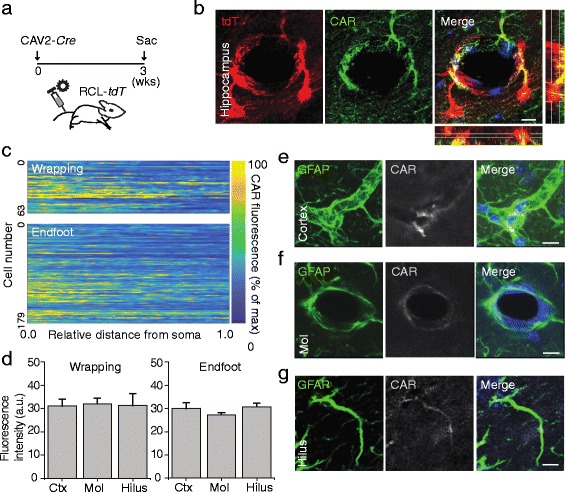
CAV2 infects CAR-expressing cells contacting the cerebral vasculature. **a** Experimental timeline of CAV2-*Cre* intravenous injection. **b** Representative images of CAR expression in tdT+ cells in the hippocampus of RCL-*tdT* mice injected with intravenous CAV2-*Cre*. **c** Heat map of CAR distribution along astrocytic cells (measured as fluorescence intensity in arbitrary units) in the hippocampus. **d** Plot of CAR density on wrapping-type astrocytes of different regions (left), One-way ANOVA *F*(2,76) = 0.001, *P* = 0.999. Plot of CAR density on endfoot-type astrocytes of different regions (right), One-way ANOVA *F*(2,212) = 1.573, *P* = 0.210. **e** Representative images of CAR expression in GFAP+ cells in cortex. **f** Representative images of CAR expression in tdT+ cells in the molecular layer of the dentate gyrus. **g** Representative images of CAR expression in tdT+ cells in the hilus of the dentate gyrus. *n* = 3–4 animals per experiment. The scale bars are 10 μm

Given the striking specific targeting of astrocytes via intravenous CAV2 injection, we wondered how the specificity could be reached. It has been shown that CAV2 infection is mediated via interaction with cell membrane-bound Coxsackie Adenovirus Receptor (CAR) [9, 57]. Indeed, the labeling and transgene delivery to neurons via stereotaxic injection of CAV2 is consistent with high expression of CAR on neurons (Additional file 1: Figure S7) and [48, 65]. We thus investigated the expression of CAR in astrocytes. We first stained sections of CAV2-*Cre*-injected brain tissue of RCL-*tdT* mice with antibodies against CAR (Fig. 3a). We observed substantial cellular expression of CAR in both types of tdT+ astrocytes (Fig. 3b and c), consistent with CAR expression in neurons (Additional file 1: Figure S7a). To exclude the possibility of nonspecific binding of the CAR-directed antibody, we evaluated this antibody by genetically overexpressing CAR in neurons via an AAV harboring a CAR fused to a GFP reporter (Additional file 1: Figure S7b). As shown in Additional file 1: Figure S7c, we observed robust CAR expression on GFP+ cells as compared to non-GFP+ cells, suggesting the antibody’s specificity to CAR.

Given the lack of CAV2-mediated labeling in the hilus (Fig. 2), we sought to determine whether the lack of labeling in the hilus was due to a lack of viral infectivity within the hilar compartment. We analyzed the CAR signal on hilar astrocytes, and compared this to the CAR signal on astrocytes of other regions. Hilar astrocytes had a similar density of CAR as compared to astrocytes in other brain regions (Fig. 3d-g). This suggests that the hilar astrocyte escape from intravenous CAV2 labeling (Fig. 3 and Additional file 1: Figure S2) did not result from a lack of CAR expression among this population of cells (Fig. 3g).

Several possibilities remain for this differential labeling: adenoviruses rely on co-receptors and adapter molecules to facilitate infection [2], which may be lacking in the hilar astrocyte. Another possibility is that components of the hilar blood supply differ from those of other brain regions where astrocytes were infected by CAV2. Given the role of astrocytes in recruiting an arterial blood supply [25], and the relatively lower association between hilar astrocytes and microvessels (Fig. 1e and Additional file 1: Figure S1), we speculated that the hilus may be enriched with venules, the draining vessels. To test this possibility, we injected the arterial-specific dye Alexa Fluor 633 hydrazide [52] into the tail veins of RCL-*tdT* mice that had been previously injected with CAV2-*Cre* to mark astrocytes (Additional file 1: Figure S8). Consistent with arterial-specific labeling of astrocytes with CAV2-*Cre*, we observed high concordance between the tdT signal and dye-labeled putative arteries and arterioles in neocortex (Additional file 1: Figure S8). Due to poor penetration of the Alexa Fluor 633 dye into the hippocampus (data not shown), we were unable to assess the possible spatial segregation between arterial and venous vessels in this region. Such an analysis will require a novel method or detailed electron microscopy.

Together, these data suggest that astrocytes express CAR, which likely mediates uptake of intravenously administered CAV2 into these cells. As described above, we noted a paradoxical preferential labeling of neurons via stereotaxic CAV2 injection (Additional file 1: Figure S2b) and preferential labeling of astrocytes via intravenous CAV2 injection (Fig. 2 and Additional file 1: Figure S2a, c, d). Intravenously-delivered CAV2 may enter astrocytes preferentially via exosomes delivered across endothelial cells, while direct injection of CAV2 into the brain parenchyma may result in direct infection of cells without the formation of exosomes, which could potentially explain this difference. However, this remains an interesting curiosity for further investigation.

### Depletion of astrocytes by targeted DTR expression

**Fig. 4:**
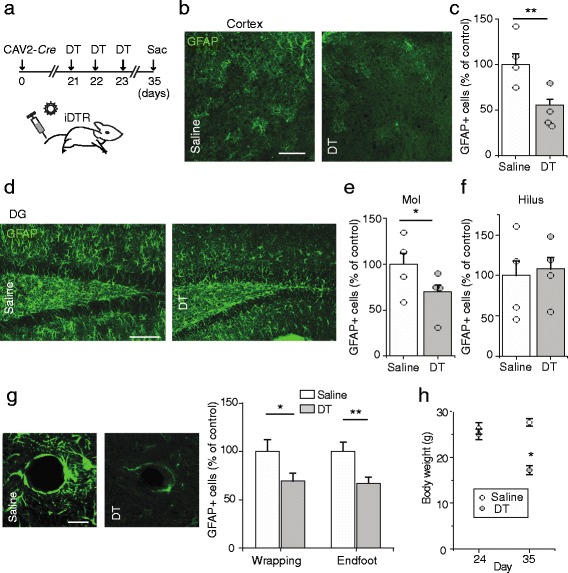
Targeted depletion of astrocytes with intravenous CAV2 injection. **a** Experimental timeline in which iDTR mice are first injected with intravenous CAV2-*Cre* to induce iDTR expression in targeted cells, then treated with intraperitoneal DT to deplete DTR+ cells. **b** Representative images of GFAP staining in layer I of cortex of CAV2-*Cre*-injected iDTR mice treated with DT or saline. The scale bar is 50 μm. **c** Plot of GFAP+ cells in the cortex of iDTR mice treated with DT or saline. Two-tailed unpaired t-test *P* = 0.0013. **d** Representative images of GFAP staining in the dentate gyrus of CAV2-*Cre*-injected iDTR mice treated with DT or saline. The scale bar is 50 μm. **e** Plot of GFAP+ cells in the molecular layer (Mol) of the dentate gyrus of iDTR mice treated with DT or saline. Two-tailed unpaired t-tests, *P* = 0.021. **f** Plot of GFAP+ cells in the hilus of the dentate gyrus of DT and saline-treated mice. Two-tailed unpaired t-test, *P* = 0.732. **g** 40× magnified representative images of GFAP staining in the hippocampus of CAV2-Cre-injected iDTR mice treated with DT or saline (left). The scale bar is 10 μm. Subgroup analysis of GFAP+ cells, segregated by wrapping-type and endfoot-type GFAP-expressing cells of iDTR mice treated with DT or saline (right). Two-tailed unpaired t-tests, *P* = 0.043, 0.007, respectively. **h** Plot of animal weights across the experiment. Two-tailed, paired t-tests, Saline group: *P* = 0.162; DT group: *P* = 0.041. Data are plotted as mean +/− SEM. * indicates P < 0.05, ** indicates P < 0.01. *n* = 4 animals per condition

We next evaluated whether the expression of intravenous CAV2-delivered transgenes would be sufficient for genetic manipulation. We injected CAV2-*Cre* into the tail veins of inducible diphtheria toxin receptor (iDTR) mice, allowing us to selectively ablate Cre-expressing cells via subsequent treatment with diphtheria toxin (DT) [11]. Three weeks after the CAV2-*Cre* injection, we administered DT or an equal volume of saline as control for 3 consecutive days (Fig. 4a). On day 35 of the experiment, we killed the animals. The density of astrocytes decreased substantially in the DT group as compared to the saline group (Fig. 4b and c). In the dentate gyrus, there was a biased decrease in astrocytes of the molecular layer, but not of the hilus (Fig. 4d-f). The number of wrapping-type cells and endfoot-type cells were both substantially decreased (Fig. 4g). To confirm the depletion was not an off-target effect of DT administration, we measured astrocyte density in DT-treated wild type animals and found comparable density with saline-treated iDTR animals (Additional file 1: Figure S9). The successful ablation of astrocytes suggests the sufficient expression of DTR mediated by intravenous CAV2 transgene delivery.

We next determined whether the depletion of astrocytes affected the overall health or survival of the animals. While we observed no change in body weight in the saline group and no animal deaths in either condition across the experiment, there was a significant decrease in weight of DT-treated animals by day 35 (Fig. 4h). This suggests that the astrocyte depletion was accompanied by a decrease in the animals’ functional status, consistent with previous findings [16, 33, 44].

Taken together, these data show that intravenous CAV2 transgene delivery provides the capability to perform non-invasive manipulation of astrocytes. This manipulation also provides us with a novel method to investigate the distinct function of this population of brain cells.

### Perivascular astrocyte depletion impaired hippocampus-dependent contextual memory

**Fig. 5:**
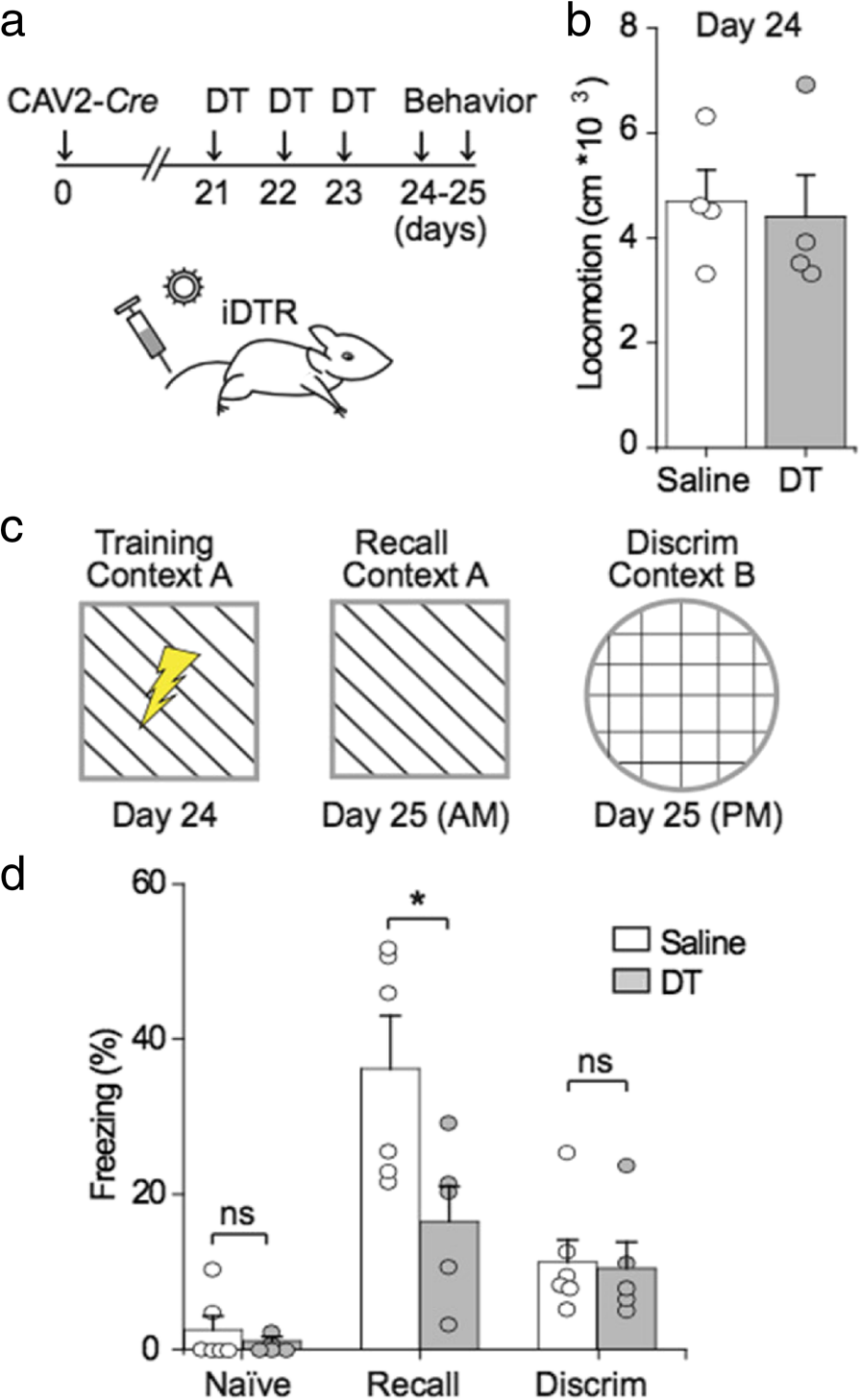
Depletion of astrocytes impairs contextual memory performance. **a** Experimental timeline, in which iDTR mice are first injected with intravenous CAV2-Cre to induce iDTR expression in targeted cells, then treated with intraperitoneal DT to deplete DTR+ cells, with behavioral tests taking place over the subsequent two days. **b** Plot of total distance covered during a 5-min recording session in a 1 m^3^ open field. Two-tailed unpaired t-test *P* = 0.783. **c** Contexual fear memory paradigm, in which mice are first exposed to a novel context A, paired with a footshock, then re-exposed to the same context A the following morning without a footshock, followed by a novel context B in the afternoon without a footshock. **d** Plot of freezing (percentage of total time in behavior chamber) in naive animals in context A on day 24, during recall in context A on day 25, and during discrimination in context B on day 25. Naive: Saline vs. DT, two-tailed unpaired t-test *P* = 0.168. Recall & Discrimination: Two-way ANOVA *F*(2,8) = 3.494, *P* = 0.081. Test of between-subjects effects, Recall: *F*(1,9) = 6.467, *P* = 0.032; Discrimination: *F*(1,9) = 0.033, *P* = 0.860. Data are plotted as mean +/− SEM. * indicates P < 0.05, ** indicates *P* < 0.01. *n* = 5–6 animals per condition

Rather than directly assessing changes in vascular activity upon astrocyte depletion, which is technically challenging especially in deep brain areas such as the hippocampus, we sought to determine whether astrocytic depletion would result in functional deficits prior to any obvious changes in body weight (Fig. 4h). Given that astrocytes in the molecular layer of the dentate gyrus may couple neuronal activity to vascular recruitment during memory-related tasks [26], we wondered whether there would be deficits in cognitive performance. We tested iDTR mice that had been intravenously injected with CAV2-*Cre* and treated with DT on the open field test and a contextual memory discrimination task as illustrated in Fig. 5a [21]. As shown in Fig. 5b, there were no differences in open field locomotion between groups. We then performed a contextual discrimination paradigm as illustrated in Fig. 5c. Briefly, mice were first exposed to a novel context A for 3 min, at the end of which they received a 3-s foot shock (0.5 mA). On the next day, in the morning, they were re-exposed to the now familiar context A for 5 min without a foot shock. In the afternoon, they were exposed to a novel context B for 5 min, again without a foot shock. Baseline (naive) freezing activity in context A did not differ significantly between groups (Fig. 5d), consistent with the lack of significant locomotion differences between groups on the open field test (Fig. 5b). However, compared to saline-treated controls, DT-treated mice exhibited significantly lower freezing in the recall context, although no significant change was observed in the discrimination context (Fig. 5d). This suggests that astrocytes may play an essential role in hippocampus-dependent contextual memory expression. However, the cellular mechanism underlying this phenotype requires further testing with this method. For example, it would be interesting to know the effects of astrocytic depletion on the blood flow rate and local circuit activity.

### Impaired survival of adult-born hippocampal neurons following the depletion of astrocytes

We further evaluated whether astrocyte depletion had an effect on brain activity-dependent processes, which rely critically on neurovascular interactions. Since the survival of adult-born neurons is impacted by vascular activity [12, 19, 28, 37, 60], we tested whether astrocyte depletion would affect the survival of adult-born DGCs.

**Fig. 6:**
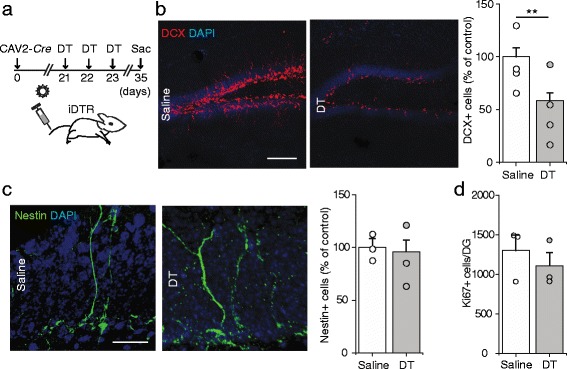
Depletion of astrocytes decreases the survival of adult-generated dentate granule neurons. **a** Experimental timeline in which iDTR mice are first injected with intravenous CAV2-Cre to induce iDTR expression in targeted cells, then treated with intraperitoneal DT to deplete DTR+ cells. **b** Representative images of DCX staining in the dentate gyrus from saline- and DT-treated iDTR mice. The scale bar is 100 μm. On the right is a plot of DCX+ cell density in the GCL of DT and saline treated iDTR mice. Two-tailed, unpaired t-test, *P* = 0.0006. **c** Representative images of Nestin + cells in the SGZ of iDTR mice treated with saline or DT. On the right is a plot of the density of Nestin + cells in the SGZ. Two-tailed unpaired t-test, *P* = 0.776. The scale bar is 10 μm. **d** Plot of Ki67+ cells per dentate gyrus of saline- and DT-treated iDTR mice. Two-tailed unpaired t-test *P* = 0.488. Data are plotted as mean +/− SEM. * indicates *P* < 0.05, ** indicates *P* < 0.01. *n* = 4 animals per condition

We injected DT for 3 consecutive days to iDTR mice 3 weeks after CAV2-*Cre* injection and measured the number of adult-born dentate granule cells (DGCs) 11 days after the last DT injection (Fig. 6a). Following astrocyte depletion, there was a dramatic decrease in the total number of immature neurons, stained with the immature neuronal marker doublecortin (DCX) (Fig. 6b). To determine whether the decrease in DCX+ cells resulted from a compromised neural stem cell pool, we analyzed the number of radial glia-like progenitors, labeled with Nestin, a neural progenitor marker [58], in the subgranular zone of the dentate gyrus, and found no change (Fig. 6c). Likewise, we observed no significant change in the number of cells positive for the proliferative marker Ki67 in the neurogenic zone, suggesting that proliferation of the progenitor cells was likely unaffected by our astrocytic depletion, at least in the tested time window (Fig. 6d). We next asked whether the depletion of astrocytes would have an effect on circuit-level neuronal activity, which may in and of itself decrease the number of DCX+ cells via alternative activity-dependent mechanisms [29, 31]. We stained brain sections from resting, unstimulated mice kept in their home cages for the activation marker c-Fos to assess basal DG network activity. As shown in Additional file 1: Figure S10, we observed no significant change in the number of c-Fos + cells in the dentate gyrus between groups, at least at this tested stage.

These experiments suggest that in the hippocampus, astrocytes might play an important role in coupling vascular recruitment and neuronal activity to affect the survival of new neurons. However, we should point out that our current method was global astrocytic ablation. To precisely test the function of neurovascular coupling, we will have to perform local astrocytic depletion, for example by focal DT application.

## Discussion

How the neurovascular unit works is poorly understood, mainly due to a shortage of tools capable of specifically targeting and manipulating its individual components in the same animal. In this study, we developed a system for targeting this cellular machinery, and in particular, the brain’s astrocytes that serve as an intermediary in this unit. In brief, using a viral screen, we identified CAV2 as a blood brain barrier permeable virus capable of noninvasively targeting astrocytes. During viral screening, we confirmed that AAV9 preferentially infected vascular endothelial cells. We further tested the efficiency of transgene delivery using our new approach. Using an iDTR mouse line, we found that intravenous CAV2-*Cre* delivery could noninvasively and specifically eliminate astrocytes from the adult brain. We have not only demonstrated that CAV/AAV Targeting of Neurons and Astrocytes Perivascularly, which we call “CATNAP,” is a tractable approach to target astrocytes of the CNS for genetic manipulation but also made several novel discoveries. For example, we found the regional heterogeneity of the glio-vascular unit and the importance of this unit for proper maintenance of the activity-dependent brain process of survival of adult-born neurons. We envision that the CATNAP method will prove valuable for future discovery of the homeostatic functioning and pathological dysfunction of neurovascular coupling and blood brain barrier function.

### Genetic dissection of the machinery of the blood brain barrier

Our understanding of the relationship between neurons and their blood supply has become enriched in recent years with a cluster of new imaging tools. For example, using two-photon imaging of somatosensory cortical microvessels in tandem with tactile stimulation of anesthetized mice, Wei et al. discovered that activity-related functional hyperemia is sensed by erythrocytes [64]. Another study that employed two-photon imaging of vessels in visual cortex during visual stimulation found robust increases in lumen dilation and blood flow velocity in tandem with calcium-induced fluorescent responses of adjacent neurons [46]. One very recent study showed capillary endothelial cells respond to increased neuronal activity via inward-rectifier K+ channels to rapidly recruit an increased blood supply via arteriolar dilation of upstream vessels [38]. Meanwhile, perivascular support cells, including not only astrocytes, but also smooth muscle cells and pericytes, have vital roles in sensing neuronal activity to regulate nutrient delivery, cerebral perfusion, and maintenance of tight junction integrity of the barrier [30, 32, 36, 45]. Whether and how astrocytes regulate these processes to affect brain metabolism and homeostatic mechanisms, as explored in the present study, remains to be tested. Previously, it was shown that intravenous AAV serotype 9 can target astrocytes during early developmental stages in rodents [10, 18], yet tools to non-invasively target astrocytes in the adult brain have been lacking. Our virus-based marking and manipulation will be able to directly target these cellular components to understand the barrier’s structural and functional properties in the adult brain.

More important, with the multitude of human diseases in which these cellular components become disrupted, including Alzheimer disease, vascular dementia, multiple sclerosis, and ischemic brain injury, this viral method to dissect the machinery may facilitate our enhanced understanding of the fundamental principles underlying the highly vulnerable system of supply and demand that exists to maintain brain homeostasis. Furthermore, this method may provide a foundation for developing genetic-based therapeutic delivery systems.

### Paradox of the specificity of viral infection and transduction

In light of the present work, we found that both astrocytes and neurons express the Coxsackie Adenovirus receptor necessary for CAV2 infection. Why, then, does intravenous CAV2 preferentially target glia but not neurons, and vice versa for stereotaxic brain injection? We speculate that viral particles may be able to specifically enter vessel-associated astrocytes via their direct contact with the adluminal side of capillaries and arterioles (Fig. 3), although more precise and high-resolution imaging will likely be needed to confirm this. The lack of astrocytic infection given stereotaxic injection is unexpected given the trafficking of CAR throughout the somatic surface of the astrocytes (Fig. 3). Perhaps there is another associated signal, which is distributed biasedly on the surface of astrocytes along the vessel. Relatedly, how does CAV2 infect perivascular astrocyte processes but seemingly fail to transduce endothelial and smooth muscle cells when delivered intravenously? We speculate that a transcytosis mechanism might be at work, though the underlying cell biology will require further elucidation.

Additionally, why are certain brain regions seemingly protected against infection from bloodborne CAV2 particles? This latter finding (Figs. 2 and 4), along with the structural diversity of vessel-associated glia (Fig. 1), suggests that the blood brain barrier may not be a homogeneous entity, but rather a functionally heterogeneous structure. In alignment with this idea, recent work has uncovered that hippocampal radial glia-like stem cells can be parsed based on morphology, fate potential, vascular connections, and proliferative activity [20, 43]. Although here, we labeled hippocampal astrocytes that were Nestin-negative and thus not radial-glia like cells, hippocampal astrocytes may exhibit a comparable degree of heterogeneity as hippocampal RGL cells, which are related in developmental lineage. Further characterization of the neuro- and glio-vascular units across brain regions will likely reveal the underpinnings of this structural and functional diversity.

### Success in depletion and possible activation or silencing

As a proof-of-principle of the CATNAP approach, we showed in Fig. 4 that astrocytes could be noninvasively targeted and ablated. We showed that genetic dissection of the glio-vascular unit resulted in severe deficits in the survival of adult-born dentate granule cells and hippocampal neurogenesis-dependent behavior. Note that the experiments presented here demonstrate the sufficiency of this method for facilitating research into neurovascular coupling. The precise mechanisms underlying these consequences, as well as functional ramifications in other brain regions, need further investigation.

How exactly might deletion of astrocytes lead to impaired cognitive function? Neurometabolic coupling, the process whereby enhanced synaptic activity leads to an upregulation of glucose supply to a local brain region [40], is likely disrupted in the face of fewer brain astrocytes. In our study, we examined hippocampus-dependent memory function upon astrocyte deletion, thus representing a window in time during which brain energy supply was likely compromised. Baseline locomotion was unaffected, suggesting that homeostatic functions of the brain may still be intact despite a significantly depressed number of functional astrocytes. However, upon increased metabolic demand, such as that required for proper hippocampal memory expression, the energy balance may be tipped such that performance is impaired as metabolic demand outstrips supply. The precise relationship between “cognitive load” and astrocyte-dependent metabolic requirements remains uncertain, but will likely be elucidated via careful behavioral experiments with titration of the percentage of astrocytes depleted or inhibited in a given brain region.

In any case, this test case that we chose raises some very interesting questions. For example, 1) why did the biased ablation of astrocytes in the dentate gyrus have such a strong impact on the survival of newborn neurons? 2) What is the function of the hilar astrocyte? 3) Will functional hyperemia be altered after astrocyte depletion? Of note, we used DTR-mediated cell ablation as one example of our approach, but we should be able to simply extend this manipulation to include chemo- and optogenetic approaches for further studies.

In summary, we have demonstrated a novel virus-based method to specifically target perivascular astrocytic cells. These dissected cells, which have been elusive to study given the scarcity of experimental tools, are likely key mediators of neuron-to-vessel communication in ways that until recently had been purely hypothetical. We expect the present method can be extensively used to label and genetically control this machinery to explore its structural and functional features in the adult brain.

## Additional file

Additional file 1: Figure S1. Comparison of neocortical and hippocampal astrocytes. **Figure S2.** Intravenous viral injection screen identified CAV2 as a tool to target non-neuronal cells throughout the central nervous system. **Figure S3.** Specificity of tdTomato expression in intravenous CAV2-labeled tdT-expressing cells. **Figure S4.** Morphological characterization of intravenous CAV2-labeled tdTomato-expressing cells. **Figure S5.** Density of blood vessels in the dentate gyrus. **Figure S6.** Three-pronged interrogation of the neuro-glio-vascular unit. **Figure S7.** Validation of Coxsackie Adenovirus Receptor expression. **Figure S8.** Preferential arterial labeling of astrocytes via intravenous CAV2. **Figure S9.** Diphtheria toxin administration did not significantly affect astrocyte density in wildtype animals. **Figure S10.** Depletion of astrocytes did not significantly affect cFos expression in the dentate gyrus. **Table S1.** Viral injection screen to identify candidate virus for astrocyte labeling. (DOCX 52436 kb)

## Abbreviations

AAV: Adeno associated virus
CAR: Coxsackie adenovirus receptor
CAV: Canine adenovirus
DGC: Dentate granule cell
GFAP: Glial fibrillary acidic protein
