# Supplementary material for: Genetic dissection of the neuro-glio-vascular machinery in the adult brain
Source: Mol Brain. 2018 Jan 15;11:2. doi: 10.1186/s13041-017-0345-4 (PMC5769320; doi:10.1186/s13041-017-0345-4)
Supplement: Additional file 1: Figure S1. — Comparison of neocortical and hippocampal astrocytes. Figure S2. Intravenous viral injection screen identified CAV2 as a tool to target non-neuronal cells throughout the central nervous system. Figure S3. Specificity of tdTomato expression in intravenous CAV2-labeled tdT-expressing cells. Figure S4. Morphological characterization of intravenous CAV2-labeled tdTomato-expressing cells. Figure S5. Density of blood vessels in the dentate gyrus. Figure S6. Three-pronged interrogation of the neuro-glio-vascular unit. Figure S7. Validation of Coxsackie Adenovirus Receptor expression. Figure S8. Preferential arterial labeling of astrocytes via intravenous CAV2. Figure S9. Diphtheria toxin administration did not significantly affect astrocyte density in wildtype animals. Figure S10. Depletion of astrocytes did not significantly affect cFos expression in the dentate gyrus. Table S1. Viral injection screen to identify candidate virus for astrocyte labeling. (DOCX 52436 kb) [file 13041_2017_345_MOESM1_ESM.docx]

**Additional file 1: Figures and Legends**

**Additional file 1: Figure S1. Comparison of neocortical and hippocampal astrocytes**

(a) Plot of astrocytic endfoot diameter in different regions. One-way ANOVA *F*(2,100) = 0.271, P = 0.763.

(b) Plot of the average number of branches per cell for astrocytes in different regions. One-way ANOVA *F*(2,206) = 6.458, P = 0.002. Post-hoc LSD tests, Mol vs Hilus, P = 0.012; Mol vs Ctx, P = 0.001; Hilus vs Ctx, P = 0.286.

(c) Sholl analysis of astrocytes in different regions. Repeated-measures ANOVA F(2,206) = 590.347, P < 0.001; LSD tests, Mol vs Hilus, P = 0.001; Mol vs Ctx, P < 0.001; Hilus vs Ctx, P = 0.094.

Data are plotted as mean +/- SEM. * indicates P < 0.05, ** indicates P < 0.01. *n* = 4 animals.

**Additional file 1: Figure S2. Intravenous viral injection screen identified CAV2 as a tool to target non-neuronal cells throughout the central nervous system**

(a) Representative images (where applicable) of fluorescent reporter expression from the various virus serotypes and administration routes.

(b) Experimental timeline for CAV2-*Cre* injection into the cortex or dentate gyrus of RCL-*tdT* mice (left). Representative images of dTom expression showing lack of co-localization with GFAP (right). The scale bar is 10 μm.

(c) Representative images of the cortex of RCL-tdT mice injected with CAV2-*Cre* intravenously at 6 weeks post injection (wpi). The scale bar is 50 μm.

(d) Representative images of the dentate gyrus of RCL-tdT mice injected with CAV2-Cre intravenously at 6 weeks post injection (wpi). On the right is a plot of dTom density in the various layers of the dentate gyrus at 6wpi. One-way ANOVA *F*(2,24) = 23.1, P < 0.001; LSD tests Mol vs GCL, P = 0.201; Mol vs Hilus, P < 0.001; GCL vs. Hilus, P < 0.001.

(e) Representative images of Cre recombinase and dTom co-localization in the hippocampal Mol and hilus. The scale bars are 50 μm.

Data are plotted as mean +/- SEM.  * indicates P < 0.05, ** indicates P < 0.01. *n* = 3-4 animals per experiment.

**Additional file 1: Figure S3. Specificity of tdTomato expression in intravenous CAV2-labeled tdT-expressing cells.**

(a) Sample image of Nestin and tdT staining in the GCL.

(b) Sample image of CC1 and tdT staining in the Mol.

(c) Sample image of PDGFRα and tdT staining in the Mol.

(d) Sample image of NG2 and tdT staining in the Mol.

(e) Sample image of Iba1 and tdT staining in the Mol.

(f) Sample image of NeuN and tdT staining in the Mol.

The scale bars are 20 μm. *n* = 4 animals.

**Additional file 1: Figure S4. Morphological characterization of intravenous CAV2-labeled tdTomato-expressing cells.**

(a) Plot of endfoot- vs. wrapping-type dTom+ cells in the cortex.

(b) Representative image of dTom+ cells and contacted CD31+ endothelial cells in the cortex. The scale bar is 10 μm.

(c) Histogram of distances between dTom+ cell bodies and contacted CD31+ cells in the cortex.

Data are plotted as mean +/- SEM. *n* = 3 animals per experiment.

**Additional file 1: Figure S5. Density of blood vessels in the dentate gyrus**

Shown on the left is a representative image of CD31+ vessels in the dentate gyrus. On the right is a plot of the relative area of each subregion occupied by blood vessels. One-way ANOVA *F*(2,46) = 0.315, P = 0.731.  *n* = 3 animals. The scale bar is 100 μm.

**Additional file 1: Figure S6. Three-pronged interrogation of the neuro-glio-vascular unit**

(a) Experimental timeline of CAV2-Cre injection and subsequent AAV-*CaMKII-GFP* into RCL-*tdT* animals (top left). Representative image taken from an iDTR mouse injected with CAV2-*Cre* (tail vein) followed by AAV-*CaMKII-GFP* into the dentate gyrus, with co-staining for CD31 to show hippocampal vasculature (top center and left panels). The inset (dashed box) is at higher magnification below.

(b) Representative image taken from a RCL-*tdT* mouse injected with CAV2-*Cre*, demonstrating the relationship between the vasculature (CD31), perivascular astrocytes (tdT), and axon terminals (SMI312) in the inner Mol of the dentate gyrus.

(c) Experimental timeline of AAV9-*CAG-GFP* tail vein injection into wildtype mice (left). Representative images of CD31+ vessel co-labeling in the Mol of the dentate gyrus (right).

The scale bar is 10 μm. *n* = 4 animals per experiment.

**Additional file 1: Figure S7. Validation of Coxsackie Adenovirus Receptor expression**

(a) Representative image of a AAV-*CaMKII*-driven *GFP*-labeling of dentate granule cells in the dentate gyrus stained for CAR. Shown below is a representative image of a retrovirally-labeled dentate granule cell co-localizing with CAR at the axon initial segment. Arrows point to regions of co-localization. The scale bar is 10 μm.

(b) Experimental timeline of AAV-*CAR-GFP* injection into auditory thalamus and CAV2-*Cre* injected into auditory striatum (unilaterally).

(c) Representative images taken from the ipsilateral and contralateral auditory striatum from mice injected unilaterally with AAV-*CAR-GFP* into auditory thalamus, and accompanying anti-CAR antibody staining. The scale bar is 10 μm. *n* = 3 mice per experiment.

**Additional file 1: Figure S8. Preferential arterial labeling of astrocytes via intravenous CAV2**

Experimental timeline of intravenous CAV2 injection and intravenous Alexa Fluor 633 hydrazide (top). Representative image of tdT labeling and Alexa Fluor 633 arterial labeling in the neocortex (bottom). Plot of Alexa Fluor 633-labeled vessels associated or not associated with wrapping tdT+ cells (right). Two-tailed unpaired t-test P < 0.001.

Data are plotted as mean +/- SEM. * indicates P < 0.05, ** indicates P < 0.01. Scale bar is 10 μm. *n* = 3 animals per experiment.


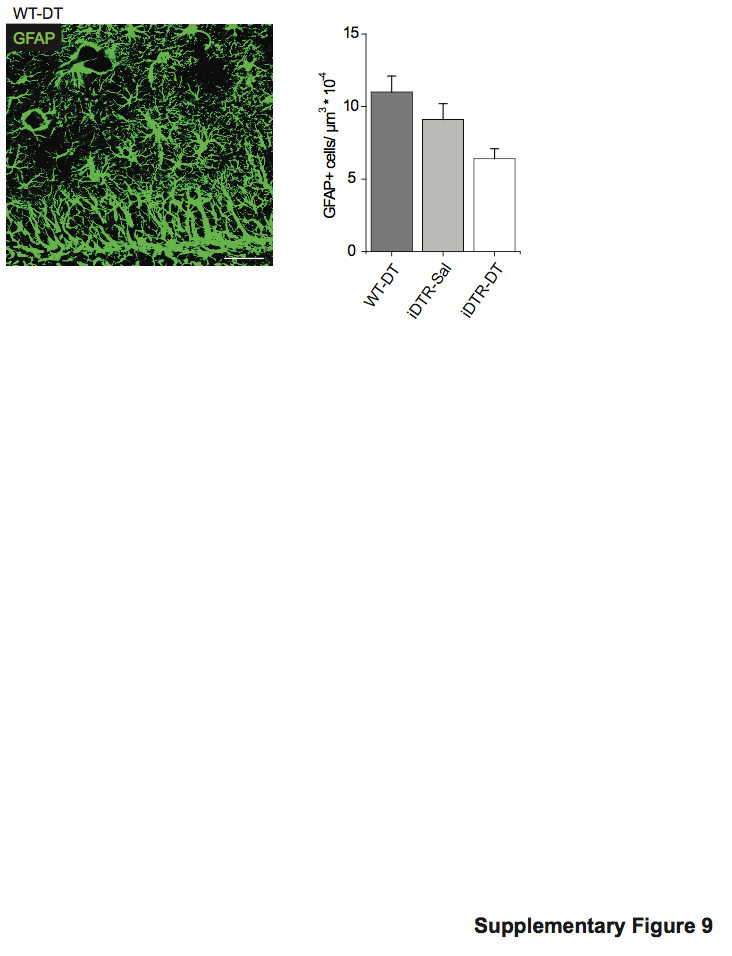


**Additional file 1: Figure S9. Diphtheria toxin administration did not significantly affect astrocyte density in wildtype animals**

Representative image of GFAP staining in the dentate gyrus (left). The scale bar is 20 μm. Plot of GFAP+ cells in wildtype mice injected with CAV2-*Cre* followed by DT (WT-DT), iDTR mice injected with CAV2-*Cre* followed by saline (iDTR-Saline), and iDTR mice injected with CAV2-Cre followed by DT (iDTR-DT) (right). One-way ANOVA *F*(2,40) = 0.614, P = 0.546.

**Additional file 1: Figure S10. Depletion of astrocytes did not significantly affect cFos expression in the dentate gyrus**

Representative images of cFos+ cells in the GCL of iDTR mice treated with saline or DT (left). Plot of the density of cFos+ cells in the GCL (right). Two-tailed unpaired t-test, P = 0.279. The scale bar is 50 μm. *n* = 4 mice per group.

**Additional file 1: Table S1. Viral injection screen to identify candidate virus for astrocytes labeling**
